# Anti-Müllerian hormone and its relationships with subclinical cardiovascular disease and renal disease in a longitudinal cohort study of women with type 1 diabetes

**DOI:** 10.1186/s40695-017-0023-9

**Published:** 2017-08-18

**Authors:** Catherine Kim, Yuanyuan Pan, Barbara H. Braffett, Valerie L. Arends, Michael W. Steffes, Hunter Wessells, Aruna V. Sarma, for the DCCT/EDIC Research Group

**Affiliations:** 1 Departments of Medicine, Obstetrics & Gynecology, and Epidemiology, University of Michigan, 2800 Plymouth Road, Building 16, Room 430W, Ann Arbor, MI 48109-2800 USA; 2 The Biostatistics Center, George Washington University, Rockville, MD USA; 3 Department of Laboratory Medicine and Pathology, University of Minnesota, Minneapolis, MN USA; 4 Department of Urology, University of Washington, Seattle, WA USA; 5 Department of Urology, University of Michigan, Ann Arbor, MI USA

**Keywords:** Ovarian reserve, Anti-Müllerian hormone, Coronary artery calcification, Type 1 diabetes

## Abstract

**Background:**

Reproductive age may be a risk factor for vascular disease. Anti-Müllerian hormone (AMH) is produced by viable ovarian follicles and reflects reproductive age. We examined whether AMH concentrations were associated with markers of subclinical cardiovascular disease (CVD) and kidney disease among women with type 1 diabetes.

**Methods:**

We performed a cross-sectional analysis of the Epidemiology of Diabetes Interventions and Complications Study**.** Participants included women with type 1 diabetes and ≥1 AMH measurement (*n* = 390). In multivariable regression models which adjusted for repeated measures, we examined the associations between AMH with CVD risk factors, estimated glomerular filtration rate, and albumin excretion ratio. We also examined whether initial AMH concentrations were associated with the presence of any coronary artery calcification (CAC) or carotid intima media thickness (cIMT).

**Results:**

After adjustment for age, AMH was not associated with waist circumference, blood pressure, lipid profiles, or renal function. Higher initial AMH concentrations had borderline but non-significant associations with the presence of CAC after adjustment for age (odds ratio [OR] 1.08, 95% confidence interval [CI] 1.00, 1.16) which were minimally altered by addition of other CVD risk factors, although women in the 3rd quartile of AMH had lower odds of CAC than women in the lowest quartile (OR 0.40, 95% CI 0.17, 0.94). After adjustment for age, higher AMH was associated with statistically significant but only slightly higher cIMT (0.005 mm, *p* = 0.0087) which was minimally altered by addition of other CVD risk factors.

**Conclusions:**

Among midlife women with type 1 diabetes, AMH has slight but significant associations with subclinical measures of atherosclerosis. Future studies should examine whether these associations are clinically significant.

**Trial registration:**

NCT00360815 and NCT00360893 Study Start Date April 1994.

## Background

Reproductive age, traditionally categorized as reproductive stage (i.e. premenopausal or postmenopausal) [1], may be a risk factor for chronic diseases including cardiovascular disease (CVD) and kidney disease apart from chronologic age. For example, an early age (less than 40 years) of menopause may increase risk of both coronary disease and stroke [2], and early age at menopause is also associated with end stage renal disease among women with type 1 diabetes [3]. Presumably, such associations are mediated through ovarian hormone effects on the vascular endothelium, or conversely through the impact of chronic conditions on ovarian function [4], although the key hormones and exact mechanisms of action are not known. A serum marker of reproductive age is anti-Müllerian hormone (AMH), a dimeric glycoprotein that is produced solely by functioning ovarian follicles [5]. AMH is a member of the transforming growth factor-beta superfamily, which encompass a broad group of receptors with endothelial activity [6], and hypothetically could affect vascular disease via these receptors. In addition, AMH has relatively minimal variations within the menstrual cycle and has recently been incorporated into the STRAW (Stages of Reproductive Aging Workshop) staging system for reproductive aging [1]. As women approach menopause, concentrations of AMH decline to undetectable values [7]. Thus, AMH has been increasingly used to predict ovarian reserve in healthy populations as well as in women experiencing assisted reproductive technology [8–11].

Cross-sectional studies suggest that lower AMH concentrations are independently associated with hypertensive disorders of pregnancy [4] as well as higher blood pressure [12, 13] and less favorable lipid concentrations [13, 14]. We have previously reported that AMH was not associated with hemoglobin A1c in the EDIC cohort [15]. Reports examining AMH and subclinical atherosclerosis are few and conflict [6, 13, 16, 17]. To our knowledge, no reports in women have examined whether AMH is associated with markers of renal function.

Women with type 1 diabetes have an increased risk of CVD and kidney disease compared to women without diabetes [18]. Whether reproductive age independently increases risk for CVD in this high-risk population is not known. The Diabetes Control and Complications Trial (DCCT) was a randomized trial that enrolled participants with type 1 diabetes in order to compare the impact of intensive vs. conventional diabetes therapy upon microvascular complications [19]. During the follow-up Epidemiology of Diabetes Interventions and Complications (EDIC) study, coronary artery calcification (CAC) and serial measures of common cIMT were assessed, as well as AMH and serial measures of CVD risk factors. Thus, we were able to examine whether AMH was associated with CVD risk factor profile and measures of subclinical atherosclerosis among women with type 1 diabetes. In addition, estimated glomerular filtration rate (eGFR) and albumin excretion ratio (AER) were evaluated serially throughout DCCT and EDIC, and we were able to examine whether AMH was associated with these markers of renal disease. As previous reports have noted a U-shaped relationship between AMH and CVD risk factors [13], our hypotheses were that extremes of AMH are associated with CVD risk factor profile and measures of subclinical atherosclerosis and renal disease among women with type 1 diabetes.

## Methods

The DCCT and EDIC studies have been described in detail (14). Briefly, the DCCT was a multicenter, randomized clinical trial designed to compare the impact of intensive and conventional diabetes treatment on the development and progression of early microvascular complications of type 1 diabetes [20]. From 1983 to 1989, 1441 patients (including 680 women) were enrolled at 29 centers. The intensive treatment regimen was designed to achieve glycemic control as close to the non-diabetic range as possible using ≥3 daily insulin injections or an insulin pump. Conventional treatment consisted of 1–2 daily insulin injections without stipulated target glucose concentrations. The DCCT included a primary prevention cohort and a secondary intervention cohort. The primary prevention cohort consisted of 726 subjects with no retinopathy, urinary albumin excretion rate < 40 mg/24 h, and diabetes duration of 1–5 years at DCCT baseline. The secondary intervention cohort consisted of 715 subjects who had non-proliferative retinopathy, urinary albumin excretion rate ≤ 200 mg/24 h, and diabetes duration of 1–15 years. Individuals were excluded if they had hypertension (defined by systolic blood pressure [SBP] levels ≥140 or diastolic blood pressure [DBP] levels ≥90 mmHg), were taking any blood pressure or lipid-lowering medications, or had a history of symptomatic ischemic heart disease or symptomatic peripheral neuropathy. Of the 680 women in the original DCCT cohort, 657 were postpubertal and premenopausal at DCCT baseline.

**Fig. 1:**
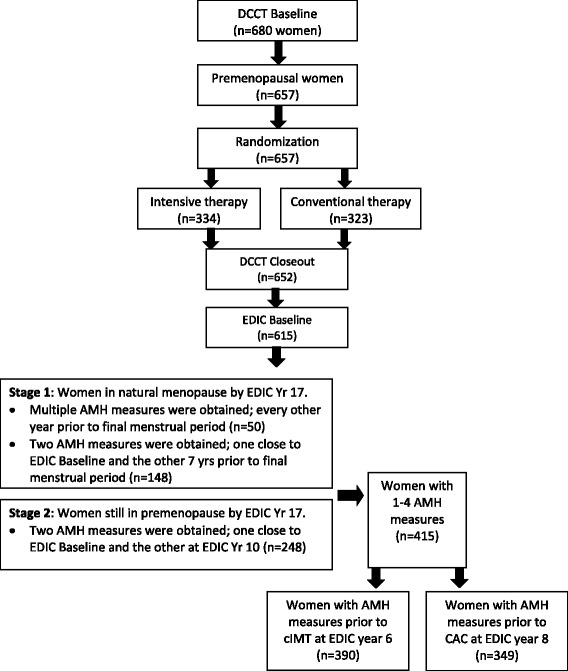
Flow chart of study participants

In 1994, 615 women from the original DCCT cohort enrolled in the Epidemiology of Diabetes Interventions and Complications (EDIC) study, designed to follow participants for long-term micro and macrovascular complications. During EDIC, a standardized annual history and physical examination included a standardized interview regarding menstrual patterns or discontinuation of menses, gynecologic surgeries, and use of exogenous sex hormones [21]. This report focuses upon the relationship between AMH, CVD risk factors, and markers of subclinical atherosclerosis during EDIC for women who had not undergone hysterectomy or oophorectomy by EDIC year 17 and also had initial serum AMH measures assessed during EDIC years 1–4, prior to CAC measures at EDIC year 8 (*n* = 349) and cIMT measures at EDIC year 6 (*n* = 390) (Fig. 1). Since AMH is made only by ovulating ovaries, it is undetectable after menopause, and previous reports noted that AMH levels were undetectable at 6 years prior to menopause [22]. We desired to limit the number of undetectable values, and so we measured AMH only prior to menopause. We also desired to have at least 2 values per woman, as AMH declines linearly prior to menopause. Therefore, for women who were naturally menopausal at EDIC year 17, AMH was assessed as close to EDIC baseline as possible and also assessed 7 years prior to their final menstrual period (*n* = 148). In a subset of these women who had reached natural menopause by EDIC year 17 (*n* = 50), AMH was measured every other year prior to menopause to ensure that the declines in AMH were linear over time. For women who were still premenopausal at EDIC year 17, AMH was assessed as close to EDIC baseline as possible and also assessed at EDIC year 10. The median number of AMH measurements per woman was 2, with an interquartile ratio of 1–4, at an average of 6.3 years apart.

Clinical and biochemical endpoints were obtained annually by history, physical exam, and laboratory testing [23]. Body mass index (BMI), waist circumference, insulin dosage, SBP and DBP, and hemoglobin A1c (HbA1c) were assessed at randomization and quarterly during DCCT and annually in EDIC [24]. Lipid profiles and urinary albumin excretion rates (AER)/glomerular filtration rates (GFR) were obtained on alternate years. Total cholesterol, triglyceride, and high-density lipoprotein cholesterol (HDL) concentrations were determined by enzymatic methods, and low-density lipoprotein cholesterol (LDL) was calculated using the Friedewald equation [25]. Medication use was assessed at each exam by EDIC staff. Cigarette use was also self-reported and smoking was defined as reporting any cigarette use currently.

AMH assays were performed on previously stored samples by the EDIC Central Biochemistry Laboratory in Minneapolis, MN using ELISA (Beckman Coulter DSL, Webster, TX second generation kit) with a quantification limit of 0.08 ng/ml [26], lower than reported for earlier generation assays [27]. Inter-assay coefficients of variation (CV) provided by the manufacturer were 8.0% at 0.15 ng/ml, 4.8% at 0.85 ng/ml and 6.7% at 4.28 ng/ml (mean = 6.5%); intra-assay CV is 4.6% at 0.14 ng/ml, 2.4% at 0.84 ng/ml and 3.3% at 4.41 ng/ml (mean = 4.0%). In the EDIC Central Biochemistry Laboratory, coefficients of variation were 8.1% at a mean concentration of 3.3 ng/ml and 4.2% at a mean concentration of 8.3 ng/ml. For values less than the 0.08 ng/ml limit of quantification but above the lower limit of detection, SoftMax Pro software (Sunnyvale, CA) was used to plot values, fit a cubic regression curve, and create splines which were then used to estimate AMH concentrations.

Carotid ultrasonography was performed at approximately 1, 6, and 12 years after the initiation of the EDIC study [28]. Carotid IMT (mm) was assessed in a central unit (Tufts University, Boston, MA) by a single reader. Computed tomography of the heart was performed once during approximately the 8th EDIC year of follow-up [28]. At that visit, participants were scanned twice over calibration phantoms of known calcium concentration with scans read centrally by readers who were masked to subject identity and previous treatment assignment. The average coronary artery CAC Agatston score from the 2 scans was used in the analysis. Presence of CAC was defined as an Agatston score > 6.25 mm^3^, which represents a value that is <1% likely to be attributable to interscan variability.

### Statistical analysis

We compared the distribution of CVD risk factors at EDIC baseline by quartile of the initial measure of AMH for each woman using analysis of variance for continuous variables and the chi-square test for categorical variables. Next, we examined whether AMH concentrations were associated with concurrent CVD risk factor values before and after adjustment for age. General linear mixed models accounted for multiple AMH measures and CVD risk factor measurements within each woman using PROC MIXED. Models examining blood pressure and renal function were additionally adjusted for anti-hypertensive medication use (angiotensin converting enzyme inhibitors, angiotensin receptor blockers, beta blockers) at the time of blood pressure measurement; other models examined lipid concentrations adjusted for lipid-lowering medication at the time of measurement. AMH was also modelled as a quadratic term, but this did not change the significance of the associations, so models are presented that assume a linear relationship between AMH and CVD risk factors.

We examined the relationship between continuous measures of AMH and presence of CAC using logistic regression models. Models used the initial AMH level during EDIC years 1–4 the primary independent variable and CAC >0 at EDIC year 8 as the dependent variable, unadjusted and adjusted for concurrent CVD risk factors measured during the same year as the initial AMH value. Due to prior studies suggesting non-linear relationships between AMH and CVD risk factors as well as non-linear relationships between AMH and CAC in unadjusted comparisons, we performed a sensitivity analysis that examined AMH in quartiles rather than as a continuous variable. Similarly, we examined the relationship between continuous measures of AMH and cIMT using linear regression models with the initial AMH level during EDIC years 1–4 as the primary independent variable and continuous cIMT measure as the dependent variable, unadjusted and adjusted for concurrent CVD risk factors measured during the same year as the initial AMH value. As these models examined only a single measure of AMH, adjustment for repeated measurements within women were not performed. All analyses were performed using SAS version 9.2 (SAS Institute, Cary, NC).

## Results

Characteristics of women at EDIC baseline by quartile of AMH value at their initial AMH measurement are shown in Table 1. Women in the lowest quartile of AMH were the oldest and women in the highest quartile of AMH were the youngest. Women in the lowest quartile of AMH had higher BMI, waist circumference, SBP, total cholesterol, LDL, and triglyceride concentrations and had lower eGFR than women in other quartiles. Duration of diabetes, cigarette use, OCP use, BMI, waist circumference, DBP, and HDL did not vary significantly by AMH quartile. Hypertension, use of hypertensive medications, and use of lipid-lowering medications was uncommon. The women in the lowest AMH quartile had the highest prevalence of CAC at year 8 (Table 2), followed by women in the highest AMH quartile, women in quartile 2, and then women in quartile 3. Although there was no relationship between quartile in AMH and cIMT at year 1, women in the lowest quartile of AMH had the highest cIMT at years 6 and year 12 followed by women in quartiles 2, 3, and 4.

**Table 1:** Participant characteristics at EDIC baseline by quartile of women’s initial AMH measurement (*n* = 390 women)

Range of AMH (ng/dl)	Quartile 1	Quartile 2	Quartile 3	Quartile 4	*p*-value
	<0.96	0.96–2.70	2.70–4.61	>4.61	
	*n* = 99	*n* = 97	*n* = 98	*n* = 97	
Age (years)	40.6 ± 6.2	34.6 ± 6.5	32.6 ± 6.0	31.0 ± 5.6	<0.0001
Diabetes duration (years)	14.4 ± 5.0	13.9 ± 5.2	14.4 ± 5.5	13.8 ± 5.4	0.78
Current smoking (%)	26.6%	20.4%	14.7%	15.1%	0.13
Current oral contraceptive use (%)	9.6%	18.3%	17.9%	11.8%	0.23
Body mass index (kg/m^2^)	26.8 ± 4.7	25.1 ± 3.8	25.8 ± 3.9	25.7 ± 3.8	0.05
Waist circumference (cm)	80.9 ± 11.2	77.2 ± 7.6	77.4 ± 8.6	78.7 ± 8.8	0.02
Systolic blood pressure (mm Hg)	118.4 ± 13.0	113.2 ± 10.8	112.4 ± 11.2	111.7 ± 12.1	0.0005
Diastolic blood pressure (mm Hg)	73.9 ± 8.9	72.8 ± 9.0	71.5 ± 8.4	72.0 ± 9.9	0.27
Hypertension^a^	10.6%	7.5%	10.5%	14.0%	0.57
Total cholesterol (mg/dl)	200.5 ± 37.6	174.4 ± 31.0	179.3 ± 30.7	189.1 ± 33.1	<0.0001
Low-density lipoprotein cholesterol (mg/dl)	123.5 ± 34.8	101.8 ± 26.2	106.1 ± 26.8	113.1 ± 26.7	<0.0001
High-density lipoprotein cholesterol (mg/dl)	59.6 ± 14.5	59.6 ± 13.6	59.7 ± 13.8	59.8 ± 14.7	0.99
Triglycerides (mg/dl)	86.5 ± 45.0	65.1 ± 28.1	66.9 ± 30.5	80.6 ± 43.4	0.0001
Lipid medication use (%)	1.1%	2.2%	0%	0%	0.29
Log albumin excretion ratio (mg/day)	2.3 ± 1.0	2.5 ± 1.1	2.2 ± 1.0	2.6 ± 1.1	0.02
Estimated glomerular filtration rate (mL/min/1.73 m^2^)	106.2 ± 11.9	114.4 ± 16.2	113.3 ± 12.5	116.7 ± 13.3	<0.0001

^a^Hypertension defined as systolic blood pressure ≥ 140 OR diastolic blood pressure ≥ 90 OR anti-hypertensive medication use

Means ± standard deviations or percentages shown

**Table 2:** Presence of coronary artery calcification (CAC) and diameter of common carotid intima media thickness (cIMT) by baseline quartile of women’s initial AMH measurement

	Quartile 1	Quartile 2	Quartile 3	Quartile 4	*p*-value
CAC > 0 (%)^a^	33.7%	17.2%	11.1%	20.5%	0.002
CIMT year 1 (mm)^b^					
Mean ± SD	0.620 ± 0.095	0.584 ± 0.059	0.595 ± 0.075	0.595 ± 0.075	
Median	0.613	0.576	0.579	0.601	
Range	(0.449–0.841)	(0.474–0.755)	(0.466–0.791)	(0.441–0.750)	0.22
CIMT year 6 (mm)^c^					
Mean ± SD	0.642 ± 0.104	0.609 ± 0.087	0.595 ± 0.086	0.596 ± 0.088	
Median	0.618	0.595	0.588	0.589	
Range	(0.451–1.10)	(0.432–0.927)	(0.429–0.824)	(0.413–0.928)	0.002

^a^Women who had AMH measures years 1–4 and CAC measurements at year 8 (*n* = 349)

^b^Women who had initial AMH measurements at EDIC year 1 and cIMT at year 1 (*n* = 172)

^c^Women who had initial AMH measurements at EDIC years 1–4 and cIMT measurements at EDIC year 6 (*n* = 390)

Means ± standard deviations or percentages shown

Table 3 shows the associations between continuous measures AMH and concurrent individual CVD risk factors before and after adjustment for age, medication use, and multiple AMH and risk factor observations per woman. Such analyses account for the fact that when there are multiple observations per woman, the resulting values are correlated i.e. cluster. In these adjusted analyses, AMH values were not associated with any CVD risk factors or measures of renal function. When AMH was modeled as a quadratic term, these associations did not change.

**Table 3:** Associations between AMH (ng/dl) and levels of concurrent cardiovascular disease risk factors and renal function (*n* = 390 women with *n* = 781 observations)

	Unadjusted	Adjusted for age and medication use^a^
Systolic blood pressure (mm Hg)	−0.32 (−0.60, −0.03)	0.005 (−0.29, 0.30)
Diastolic blood pressure (mm Hg)	−0.01 (−0.21, 0.18)	0.11 (−0.10, 0.31)
Total cholesterol (mg/dl)	−0.21 (−0.95, 0.53)	−0.02 (−0.81, 0.77)
Low-density lipoprotein cholesterol (mg/dL)	0.02 (−0.63, 0.66)	0.02 (−0.66, 0.71)
High-density lipoprotein cholesterol (mg/dL)	−0.14 (−0.44, 0.16)	−0.05 (−0.37, 0.28)
Triglycerides (mg/dL)	−0.25 (−1.22, 0.73)	−0.10 (−1.16, 0.85)
Log albumin excretion ratio (mg/day)	0.02 (−0.003, 0.04)	0.01 (−0.01, 0.04)
Estimated glomerular filtration rate (mL/min/1.73 m^2^)	0.57 (0.25, 0.88)	0.02 (−0.29, 0.34)
Waist circumference (cm)	−0.11 (−0.34, 0.12)	−0.05 (−0.29, 0.20)

^a^Antihypertensive medication use for systolic and diastolic blood pressure, log AER, eGFR, and lipid-lowering medication use for total cholesterol, LDL, HDL, and triglycerides. Age was the only adjustment made in the waist circumference model

Beta-coefficients and 95% confidence intervals shown from repeated measures regression models. Each beta-coefficient is per 1 ng/dl increment in AMH

There were 349 women who had AMH measurements during years 1–4 and CAC measurements at EDIC year 8. Table 4 shows the odds of having any CAC by AMH, before and after adjustment for age and other CVD risk factors. After adjustment for age, AMH had non-significant borderline associations with any CAC which was not altered after adjustment for covariates. In a sensitivity analysis, and due to the U-shaped relationship with prevalence of CAC in unadjusted analyses, we also examined the relationship between AMH in quartiles and the presence of any CAC. Compared to women in the lowest quartile of AMH, women in the 3rd or highest quartile of AMH had a slightly lower odds of any CAC after adjustment for age (odds ratio 0.40, 95% confidence interval [CI] 0.17, 0.94) which was similar in models which adjusted for both age and systolic blood pressure (OR 0.40, 95% CI 0.17, 0.94) and age and hemoglobin A1c (OR 0.40, 0.17, 0.94) although attenuated in models that examined age, total cholesterol, and high-density cholesterol (OR 0.46, 95% CI 0.19, 1.13). These results did not differ when treatment group was added as a covariate (data not shown).

**Table 4:** Association between initial measurement of AMH (ng/dl) during years 1–4 and CAC at year 8 before and after adjustment for concurrent CVD risk factors (*n* = 349 women)

	Unadjusted	Adjusted for age	Adjusted for age and SBP	Adjusted for age, total cholesterol, and HDL	Adjusted for age and A1c
AMH	1.01 (0.94–1.08)	1.08 (1.00–1.16)	1.08 (1.00–1.16)	1.08 (1.00–1.17)	1.08 (1.00–1.16)

Odds ratios and 95% confidence intervals shown

The association between AMH and cIMT at years 1 and 6 are shown in Table 5. For cIMT measured at year 1, AMH was not associated with cIMT at year 1 before or after adjustment for age. For cIMT measured at year 6, AMH was associated with statistically significant although only very slightly higher cIMT which was not altered after adjustment for CVD risk factors. In a sensitivity analysis, when we examined AMH in quartiles, there was no association between quartile of AMH and degree of cIMT at year 1 or degree of cIMT at year 6. Quartile of AMH was not associated with change in cIMT between EDIC years 1 to 6 (results not shown), nor was quartile of AMH associated with change in cIMT between EDIC years 6 to 12 (results not shown).

**Table 5:** Associations between initial AMH (ng/dl) during years 1–4 and cIMT (mm) before and after adjustment for concurrent CVD risk factors

	Unadjusted	Adjusted for age	Adjusted for age and SBP	Adjusted for age, total cholesterol, and HDL	Adjusted for age and A1c
Models for year 1 cIMT^a^					
AMH	0.00009 ± 0.002	0.002 ± 0.002	0.002 ± 0.002	0.002 ± 0.002	0.002 ± 0.002
* p*-value	0.95	0.11	0.10	0.22	0.13
Models for year 6 cIMT^b^					
AMH	0.00008 ± 0.002	0.005 ± 0.002	0.005 ± 0.002	0.004 ± 0.002	0.004 ± 0.002
* p*-value	0.66	0.0087	0.008	0.019	0.014

^a^Women who had AMH measurement at year 1 and cIMT measurements at EDIC year 1 (*n* = 172)

^b^Women who had AMH measurement at year 1 and cIMT measurements at EDIC year 6 (*n* = 390)

Least square means and standard errors shown

## Discussion

Women with type 1 diabetes have a higher prevalence of reproductive disorders compared to women without diabetes, including irregular menses [29], subfertility [30], and possibly polycystic ovary syndrome [31]. In addition, women with type 1 diabetes are at greater risk for CVD and renal disease than women without diabetes [18]. Thus, among women with type 1 diabetes, it plausible that reproductive dysfunction could play a role in risk of CVD and/or renal disease. AMH is increasingly used in the diagnosis and management of reproductive disorders and for reproductive staging [9]. In this cohort of women with type 1 diabetes, AMH was not associated with CVD risk factors after adjustment of age. After adjustment for age, there were associations between AMH and CAC as well as between AMH and cIMT, although the associations, when present, were slight and of unclear clinical significance. AMH was associated with significantly although minimally higher cIMT. Women who had intermediate AMH concentrations, i.e. AMH concentrations that were neither in the lowest nor the highest quartile, had the lowest odds of CAC compared to women in the lowest quartile of AMH.

Previous studies have suggested that AMH, a proxy for reproductive age, might correlate with CVD risk factors independent of chronologic age. Only one report has examined women with type 1 diabetes. In another cohort of 150 premenopausal women with type 1 diabetes, higher concentrations of AMH were associated with lower levels of SBP before and after adjustment for age [13]. There was a U-shaped relationship between AMH and HDL, where extremes of HDL were associated with low AMH concentrations. As with the current report as well as our previous report examining HbA1c and AMH [15], there was no significant correlation between AMH and DBP, AMH and LDL, or between AMH and other diabetes characteristics including diabetes duration, HbA1c, or BMI after adjustment for age. We may not have found an association between AMH and SBP due to differences between the study populations. Although both studies performed AMH and CVD risk factor measurements at similar ages, EDIC did not exclude women with histories of irregular menses, and it is possible that the relationship between AMH and SBP differs among regularly cycling women. We also adjusted for the use of anti-hypertensive medications, although this should have accentuated any existing associations between AMH and blood pressure. We were also able to examine multiple AMH measurements and CVD risk factor measurements within each woman over time in a larger population.

Studies of AMH and CVD risk factors in populations without diabetes conflict, although the associations between AMH and CVD risk factors, when documented, have been modest. Reports in populations without diabetes have noted that AMH concentrations were not cross-sectionally associated with blood pressure or lipid profiles after adjustment for age and BMI [12, 32]. Tehrani et al. reported that women in the lowest quartile of AMH eventually had slightly greater increases in total cholesterol and LDL over a decade compared to women in other quartiles [14]. However, adjustment for medication use was not noted, and the increases were slight, the equivalent of 0.39 mg/dl per year, and adjustment for age was performed by transforming actual AMH levels into age-specific AMH levels, which might have minimized the impact of age-adjustment. AMH was not associated with progression in other risk factors; to our knowledge, previous reports have not examined whether AMH is associated with renal function.

Whether AMH could subsequently increase risk of atherosclerosis via CVD risk factors or through an independent pathway has not been well-studied. One report in macaques (*n* = 66) suggested that AMH was associated with atherosclerotic plaque size [16]. Macaques in the lowest tertile of AMH had greater plaque area than macaques in the highest tertile. Similarly, Looby et al. [17] noted that in a cohort consisting of HIV positive and negative women, postmenopausal women had higher prevalence of coronary plaque vs. perimenopausal women vs. premenopausal women. AMH concentrations were undetectable in the first two groups and detectable in the last group. Other adjustments were not performed, so the association may have been due to other factors characterizing the menopausal transition. Our report suggests that if AMH and odds of CAC are associated, the relationship is not linear. Women in the highest quartile of AMH may have similar risk of CAC compared to women in the lowest quartile of AMH. While explanations are speculative, it is possible that elevated values of AMH correspond with other disorders, such as polycystic ovary syndrome, which increase the risk of CVD, and women with low values of AMH have minimal ovarian reserve, which may also increase the risk of CVD. Although mechanisms are speculative, AMH has been linked with molecules with endothelial activity, including vascular endothelial growth factor (VEGF) [33]. VEGF regulates angiogenesis, and extremes of VEGF confer decreased CVD risk [34]. Administration of VEGF has been demonstrated to stimulate AMH receptor 2 expression, which in turn can increase AMH binding in animal models [33]. Thus, AMH may be a marker for angiogenesis dysregulation. Alternatively, higher concentrations of inflammatory and endothelial dysfunction markers among lymphoma patients including interleukin 6 are significantly correlated with decreased AMH concentrations [35] as well as CVD risk [36], suggesting that AMH may also be linked with CVD through this pathway.

The two studies examining the relationship between AMH and cIMT conflict. Among women with type 1 diabetes, AMH and cIMT and between AMH and other measures of vascular flow were not related [13]. In a population of women without diabetes [6], Figueroa-Vega et al. lower AMH concentrations were strongly associated with thicker cIMT in 60 postmenopausal women. In that report, we note that the majority of postmenopausal women in this report had detectable and even elevated concentrations of AMH exceeding 5 ng/dl, despite having undergone natural menopause an average of 5 years before enrollment and use of an AMH assay with a high detection limit (0.375 ng/ml), contrary to previous reports suggesting that AMH declines to undetectable concentrations before the final menstrual period [7, 8, 14, 37, 38]. We found that that higher concentrations of AMH were associated with slightly thicker cIMT even after adjustment for covariates, although the magnitude of the association was so slight as to render clinical significance questionable.

Strengths of this report include its well-phenotyped population of women with type 1 diabetes, enabling examination of AMH with CVD risk factors as well as CAC, cIMT, and renal function. We were able to assess whether AMH was assessed with progression in cIMT as well as single measures of cIMT. As our report included women with type 1 diabetes, our results may not extend to women without diabetes, who are at lower CVD risk. We have previously reported that type 1 diabetes is associated with lower AMH concentrations [39], and thus diabetes may have modified the relationship between AMH and CVD risk factor severity and between AMH and atherosclerosis. Women in EDIC represent a cohort of a randomized trial population and experienced excellent glycemic control, and thus may not represent a more generalized population of persons with type 1 diabetes. It is possible that ovarian reserve may have stronger associations with subclinical atherosclerosis in a population with a higher severity of disease; despite their increased CVD risk from type 1 diabetes, women were in their forties at the time of their CAC and cIMT assessment, and the burden of disease was low. Similarly, end-stage renal disease was uncommon in the EDIC cohort [40], and it is possible that the relationship between AMH and renal disease would have been more pronounced in a population with a higher incidence of disease. Finally, we note that due to our sampling strategy of AMH measures prior to menopause, our power was limited to detect relationships between lower concentrations of AMH with greater burden of subclinical atherosclerosis or adverse risk factors.

## Conclusions

Among women with type 1 diabetes, AMH has minimal associations with CVD risk factors apart from chronologic age, but AMH may have a non-linear relationship with CAC and associations with cIMT. Future investigations should replicate these findings between AMH, particular in larger populations without diabetes. The clinical significance of these associations is not known and should be corroborated with investigations of outcomes. The role of reproductive stage in CVD risk has been controversial [41]. Reproductive stage is also strongly associated with other end-organ complications, including bone disease [42] and urologic conditions such vaginal atrophy [43], and investigation of the role of ovarian reserve in these complications should also be conducted. Future investigations should also explore whether the reproductive abnormalities experienced by women with type 1 diabetes are CVD risk factors apart from ovarian markers such as AMH.

## Abbreviations

AER: Albumin excretion ratio
AMH: Anti-Müllerian hormone
BMI: Body mass index
CAC: Coronary artery calcification
CIMT: Carotid intima media thickness
CVD: Cardiovascular disease
DBP: Diastolic blood pressure
DCCT: Diabetes Control and Complications Trial
EDIC: Epidemiology of Diabetes Interventions and Complications
eGFR: Estimated glomerular filtration rate
HDL: High-density lipoprotein cholesterol
LDL: Low-density lipoprotein cholesterol
SBP: Systolic blood pressure
